# Efficacy and safety of endoscopic intraperitoneal subserosal dissection for gastric submucosal tumors with extraluminal growth pattern

**DOI:** 10.1055/a-2573-9069

**Published:** 2025-05-15

**Authors:** Li-Yun Ma, Ke-Yi Guo, Xin-Yang Liu, Meng-Jiang He, Wei-Feng Chen, Sheng-Li Lin, Yao-Yao Gong, Ping-Hong Zhou, Jian-Wei Hu

**Affiliations:** 1 Endoscopy Center and Endoscopy Research Institute, Shanghai Collaborative Innovation Center of Endoscopy, Zhongshan Hospital, Fudan University, Shanghai, China; 2 Department of Gastroenterology, Jiangsu Province Hospital and Nanjing Medical University First Affiliated Hospital, Nanjing, China

## Abstract

**Background:**

Endoscopic intraperitoneal subserosal dissection (EISD) has been reported to be a promising modified tunneling technique for gastric submucosal tumors (SMTs) with a predominantly extraluminal growth pattern. We evaluated the safety and efficacy of EISD for gastric extraluminal SMTs.

**Methods:**

We prospectively enrolled consecutive patients who underwent EISD for gastric extraluminal SMTs between October 2018 and March 2024. Clinicopathological characteristics and procedure-related parameters were analyzed.

**Results:**

10 patients with 11 gastric extraluminal SMTs were included. The mean (SD) longest and shortest specimen diameters were 2.1 (0.9) cm (range 0.5–4.0) and 1.8 (0.7) cm (range 0.4–3.0), respectively. All SMTs (100%) were resected en bloc, and 10 (90.9%) were retrieved en bloc. The mean (SD) resection and suture times were 58.9 (31.5) minutes (range 26–125) and 12.4 (10.1) minutes (range 3–25), respectively. Two patients experienced type I mucosal injury at the tumor site, and no major adverse events occurred. The mean postoperative hospital stay was 3.4 (SD 0.8) days (range 3–5). No recurrence or metastasis occurred during a mean follow-up period of 32.1 (SD 18.8) months (range 7–71).

**Conclusions:**

EISD appeared to be a feasible and safe method for removing gastric extraluminal SMTs in selected patients.

## Graphical Abstract

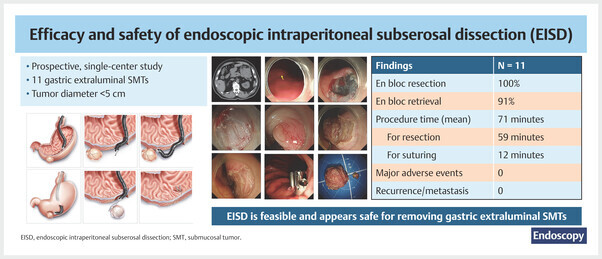

## Introduction

Submucosal tumors (SMTs), characterized by being covered with normal digestive mucosa, are most frequently found in the stomach 1 2 . Most SMTs are asymptomatic, but some, such as gastrointestinal stromal tumors (GISTs), have malignant potential. Complete surgical resection is still recognized as the primary and most important method for the treatment of gastric SMTs and for obtaining a clear pathological diagnosis 3 .

Minimally invasive endoscopic resection has attracted increasing attention recently 4 5 6 . However, for most extraluminal SMTs, the lesions are usually orientated tangentially during endoscopic dissection using traditional endoscopic techniques. The endoscopic view from the gastric lumen is inherently poor, as only a very small part of the tumor can be seen from inside. In addition, the highly movable tumor with limited endoscopic exposure increases the difficulty of en bloc resection and safe hemostasis 7 .

To overcome these disadvantages, we have developed a modified tunneling technique, endoscopic intraperitoneal subserosal dissection (EISD), for the removal of gastric extraluminal SMTs 7 . In this study, we prospectively enrolled patients with gastric extraluminal SMTs who underwent EISD and assessed the efficacy and safety comprehensively.

## Methods

### Patients

We prospectively enrolled consecutive patients who underwent EISD for gastric extraluminal SMTs between October 2018 and March 2024 at Zhongshan Hospital, Fudan University, Shanghai, China. The inclusion criteria were: 1) gastric SMTs with a growth pattern characterized by >75% of the cross-sectional diameter being located outside of the gastrointestinal lining; 2) tumor with a cross-sectional diameter of <5 cm; 3) no evidence of metastatic lesions detected on preoperative computed tomography; 4) no history of abdominal surgery. Exclusion criteria were cardiopulmonary diseases that contraindicated general anesthesia or coagulation disorders.

The study was approved by the Ethics Committee of the Zhongshan Hospital, Fudan University (B2022–095), and informed consent was obtained from all patients for all procedures and interventions.

### Procedures

**Fig. 1:**
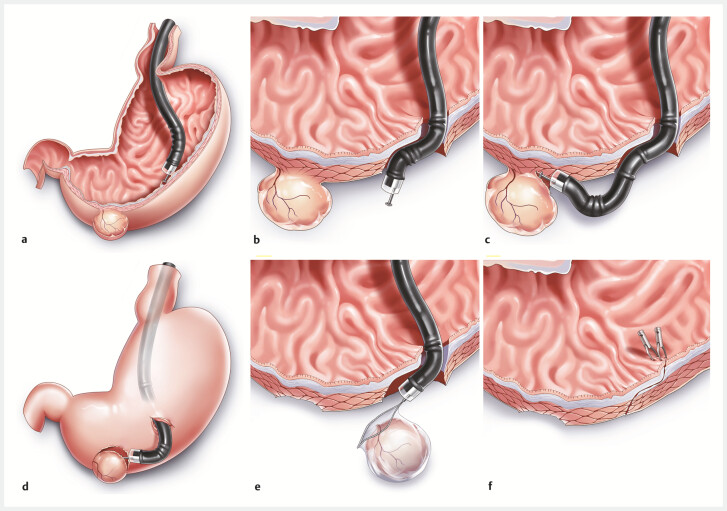
Stages of the endoscopic intraperitoneal subserosal dissection procedure. **a** Mucosal incision. **b** Submucosal tunneling and full-thickness myotomy. **c** Intraperitoneal subserosal dissection. **d** Endoscopic intraperitoneal subserosal dissection. **e** Tumor retrieval. **f** Mucosal closure.

All patients were under general anesthesia with endotracheal intubation. A 20-gauge needle was inserted into the right lower quadrant for abdominal decompression, in case of pneumoperitoneum. Details of the procedure are shown in Fig. 1 and have been reported previously 7 .

1. Mucosal incision: after submucosal injection, a 1.5-cm mucosal incision was created about 4 cm proximal to the lesion.
2. Submucosal tunneling: a short tunnel was established between the gastric submucosa and the muscularis propria, and then full-thickness myotomy was performed about 3 cm away from the lesion.
3. Intraperitoneal subserosal dissection: after locating the lesion on the serosa of the gastric wall from the abdominal cavity, the tumor was carefully dissected from the serosa and the underlying muscularis propria to avoid damaging the gastric mucosa and to keep the tumor capsule intact. Subserosal injection was performed when necessary to create working space or to identify the layers.
4. Tumor retrieval: after resection, the tumor was extracted through the submucosal tunnel to the gastric cavity and retrieved perorally by a snare.
5. Mucosal closure: after careful hemostasis, the tunnel entrance was closed with clips. A nasogastric tube was placed routinely for depression and monitoring of active bleeding.

### Postprocedural monitoring

Postoperative antibiotics and proton pump inhibitors were applied intravenously for the first 48 hours. All patients remained fasting for the first postoperative day, and if they presented no signs of active bleeding, abdominal pain, or fever, the nasogastric tube was removed and a liquid diet was administered on postoperative Day 2. Oral intake was gradually increased to a normal diet over the next 2 weeks if no adverse events occurred (e.g. fever, dyspnea, chest pain, abdominal pain).

### Histopathology

The tumor size was recorded after resection and the specimens were fixed in a 10% formalin solution for subsequent histopathological evaluation. Risk classification was estimated according to the revised National Institutes of Health grading system, which classified GISTs as very low risk, low risk, intermediate risk, and high risk based on tumor size and mitotic count 8 .

### Outcome definitions and statistical analysis

Technical success was defined as the completion of EISD without abortion or conversion to surgery. En bloc resection was defined as resection of the tumor with the capsule intact. En bloc retrieval was defined as an en bloc-resected tumor with an intact tumor capsule retrieved perorally. Resection time referred to the time from mucosal incision to lesion retrieval. Suture time was defined as the time from first metal clip insertion to complete closure of the mucosal defect. Procedure time was defined as the sum of resection time and suture time. Adverse events included procedure-related bleeding (hemoglobin drop >2 g/dL and/or need for transfusion 9 ), mucosal injury (including type I and II injuries 10 ), symptoms suggesting peritonitis (severe abdominal pain, abdominal rigidity, rebound tenderness), febrile episode (>38.5°C), and conversion to emergency surgery.

Commercial software (IBM SPSS Statistics 20; IBM Corp., Armonk, New York, USA) was used for statistical analysis. The results are expressed as mean (SD) and range or number (percentage).

## Results

### Clinicopathological characteristics

A total of 10 consecutive patients (8 males and 2 females) with 11 lesions were enrolled. The mean age was 60.4 (SD 14.1) years (range 37–77) ( Table 1 ). The mean (SD) longest and shortest diameters of specimens were 2.1 (0.9) cm (range 0.5–4.0) and 1.8 (0.7) cm (range 0.4–3.0), respectively. Ten tumors (90.9%) were located in the gastric body: five (45.5%) on the greater curvature side, two (18.2%) on the lesser curvature side, two (18.2%) on the posterior wall, and one (9.1%) on the anterior wall. The remaining tumor (9.1%) was positioned on the lesser curvature of the gastric antrum ( Table 2 ).

**Table:** **Table 1** Characteristics of 10 patients with gastrointestinal stromal tumors treated by endoscopic intraperitoneal subserosal dissection.

	N = 10
Age, mean (SD), years	60.4 (14.1)
Sex, male/female, n	8/2
Technical success, n (%)	10 (100)
Suture method, n (%)	
Metal clips	5 (50.0)
Purse-string suture	4 (40.0)
Rubber band traction-assisted closure	1 (10.0)
Adverse events, n (%)	
Bleeding ^1^	0
Peritonitis	0
Febrile episode (>38.5°C)	0
Emergency surgery	0
Mucosal injury	2 (20.0)
Postoperative peak temperature, mean (SD), °C	37.0 (0.5)
Postoperative hospital stay, mean (SD), days	3.4 (0.8)
Follow-up, mean (SD), months	32.1 (18.8)
Recurrence, n	0
Metastasis, n	0
^1^ Bleeding defined as hemoglobin drop >2 g/dL and/or need for transfusion.	

**Table:** **Table 2** Clinicopathological characteristics of 11 gastrointestinal stromal tumors treated by endoscopic intraperitoneal subserosal dissection. ^1^

	N = 11
Tumor size, mean (SD), cm	
Longest diameter	2.1 (0.9)
Shortest diameter	1.8 (0.7)
Tumor location, n (%)	
Greater curvature of the gastric body	5 (45.5)
Lesser curvature of the gastric body	2 (18.2)
Posterior wall of the gastric body	2 (18.2)
Anterior wall of the gastric body	1 (9.1)
Lesser curvature of the gastric antrum	1 (9.1)
Procedure	
Distance from perforation to lesion, mean (SD), cm	3.0 (1.0)
En bloc resection, n (%)	11 (100)
En bloc retrieval, n (%)	10 (90.9)
Procedure time, mean (SD), minutes	71.3 (37.4)
Resection time	58.9 (31.5)
Suture time	12.4 (10.1)
No. of clips, mean (range), n	6.8 (3–12)
Pathology, n (%)	
GIST	11 (100)
NIH risk stratification, n (%)	
Very low risk	6 (54.5)
Low risk	2 (18.2)
Intermediate risk	3 (27.3)
High risk	0
GIST, gastrointestinal stromal tumor; NIH, National Institutes of Health. ^1^ There were 11 lesions in total. One patient had two extraluminal tumors: one located in the greater curvature of the gastric body, and another located at the posterior wall of the gastric body.	

### Procedure-related results

**Fig. 2:**
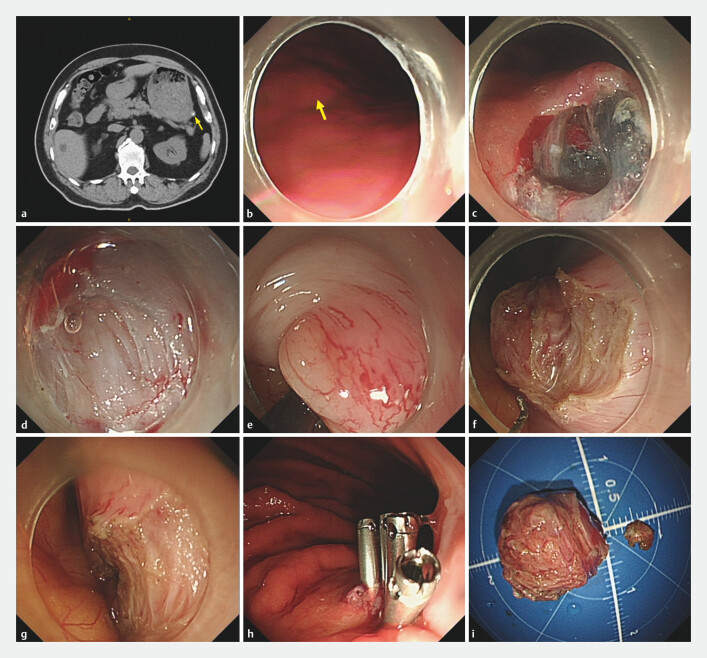
The endoscopic intraperitoneal subserosal dissection procedure. **a** Preoperative abdominal computed tomography showed a lesion with an extraluminal growth pattern in the greater curvature of the gastric body. **b** Endoscopic view of the tumor (arrow) in the gastric cavity. **c** Mucosal incision and submucosal tunneling. **d** Full-thickness myotomy. **e** Intraperitoneal view of the lesion. **f** Intraperitoneal subserosal dissection. **g** Serosal defect after resection. **h** Tunnel defect closure. **i** Resected specimens.

Technical success was 100%. The mean distance from the perforation to the lesion was 3.0 (SD 1.0) cm (range 2–5). All tumors (100%) were resected en bloc and 10 tumors (90.9%) were retrieved en bloc. One large tumor (4.0 × 3.0 cm) was retrieved in three pieces in the gastric cavity because it was difficult to retrieve through the cardia in one piece. Notably, for the patient with two tumors, both tumors were removed through one tunnel. The mean resection time was 58.9 (SD 31.5) minutes (range 26–125), and the mean suture time was 12.4 (SD 10.1) minutes (range 3–25). The tunnel entrance was closed with clips in five patients (50.0%), and purse-string suture with nylon strings was used in four patients (40.0%) because of large mucosal defects 11 . A rubber band was applied in one patient (10.0%) to assist upward traction 12 . The median number of clips used to close the tunnel was 6.8 (range 3–12) ( Table 2 , Fig. 2 ).

### Adverse events and follow-up

No procedure-related bleeding, peritonitis, febrile episode, or emergency surgery conversion occurred ( Table 1 ). Two patients experienced type I mucosal injury at the tumor site: one was managed by metal clip closure, and the other was closed with purse-string suture. The mean highest temperature after the procedure was 37.0 (SD 0.5) °C, and the mean postoperative hospital stay was 3.4 (SD 0.8) days (range 3–5).

**Fig. 3:**
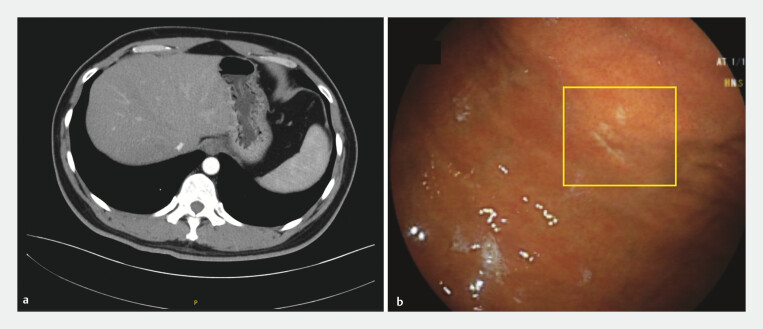
Follow-up imaging and repeat endoscopy 2 years after the procedure. **a** Follow-up abdominal contrast computed tomography. **b** Follow-up endoscopic view of previous lesion location.

Histopathological examination showed that all lesions were GISTs ( Table 3 ). According to the National Institutes of Health risk stratification, six tumors (54.5%) were classified as very low risk, two (18.2%) as low risk, and three (27.3%) as intermediate risk ( Table 2 , Table 3 ). The mean follow-up time was 32.1 (SD 18.8) months (range 7–71). No recurrence or metastasis occurred during the follow-up period ( Fig. 3 ).

**Table:** **Table 3** Details of patients and resected gastric submucosal tumors.

Patient	Sex/age, years	Tumor size, cm	En bloc resection	Procedure time, minutes	Pathology	Risk stratification	Mitotic rate, /50 HPFs	Postoperative hospital stay, days
1	M/77	2.5 × 2.0	Yes	60	GIST	Very low	1	3
2	M/37	2.5 × 2.5	Yes	70	GIST	Intermediate	10	3
3	M/70	1.6 × 1.0	Yes	68	GIST	Very low	1	5
4	F/69	2.0 × 1.8	Yes	45	GIST	Very low	3	3
5	M/58	2.0 × 2.0	Yes	40	GIST	Intermediate	6	3
6	M/76	1.3 × 1.2; 0.5 × 0.4	Yes	80	Both GISTs	Both very low	1	3
7	M/40	2.5 × 2.0	Yes	50	GIST	Intermediate	6	3
8	M/53	4.0 × 3.0	Yes ^1^	150	GIST	Low	1	3
9	F/56	2.5 × 2.0	Yes	30	GIST	Low	1	5
10	M/68	2.0 × 1.8	Yes	120	GIST	Very low	1	3
F, female; GIST, gastrointestinal stromal tumor; HPF, high-power field; M, male. ^1^ The tumor was resected en bloc but retrieved in three pieces because it was too large to retrieve through the cardia in one piece.								

## Discussion

This extended transmural endoscopic dissection technique, EISD, is a modified tunneling technique for removing gastric SMTs that have a predominately extraluminal growth pattern 7 . Our results demonstrated that EISD is effective and safe for resecting these gastric extraluminal SMTs, with no major adverse events and no recurrence or metastasis observed during a mean follow-up period of 32 months. Therefore, EISD may be a promising technique for the management of these gastric extraluminal SMTs.

Given the specific anatomical and physiological characteristics of the stomach, such as its large and nonlinear lumen, nonfixed position, and high flexibility, establishing a submucosal tunnel is technically challenging compared with in the esophagus 13 . Therefore, not all locations are suitable for submucosal tunnel creation 14 15 . Our study indicates that short tunnels into the abdominal cavity can be established from all walls of the gastric body, but this should be performed by experienced endoscopists and preceded by careful preoperative discussions to determine the location of the lesion and the tunnel site.

Our prior publication showed favorable safety and feasibility of EISD in two cases 7 . Based on our experience, proximal (oral side) incisions are easier to manage than distal (anal side) ones. For SMTs that do not have significant protrusions within the gastric lumen, metal clips can be used for localization during preoperative endoscopic ultrasound examination. Positioning the full-thickness incision approximately 3 cm away from the lesion provides a crucial working space, enabling better visualization and dissection of the lesion 7 .

En bloc resection was achieved in all patients, with a mean resection time of 58.9 (SD 31.5) minutes, which was comparable to our previous mean resection time of 60.4 (SD 37.5) minutes for resecting extraluminal SMTs using submucosal tunneling endoscopic resection (STER) 16 . We believe that the resection time can be shortened further as experience increases. En bloc retrieval was achieved in 90.9% of the tumors. Piecemeal removal was required in one patient because the tumor size prevented it from passing smoothly through the cardia; after closing the mucosal incision, the tumor was cut into three pieces in the gastric cavity. This reminds us that there is a tumor size limitation with EISD, and an optimal tumor size of under 3 cm is preferable for smooth removal. Similarly, our previous research also suggested that the implementation of STER for SMTs with a transverse diameter ≤3.5 cm may facilitate a high en bloc resection rate 17 .

Our EISD procedure offers significant advantages compared with surgical methods, including minimal trauma, fewer complications, faster recovery, lower costs, and the absence of skin incisions 7 15 . Compared with other endoscopic resection techniques, it also boasts notable advantages. First, the intact mucosa at the lesion site and the short tunnel can reduce infections and other complications resulting from full-thickness perforations. Second, compared with the uneven full-thickness defects caused by endoscopic full-thickness resection (EFTR), the tunnel makes it easier to close the mucosal defect. Importantly, the distance between the perforation and the lesion allows direct, full exposure of the lesion from the abdominal cavity, rather than from a tangential view within the gastric cavity or submucosal tunnel. This provides crucial safety for comprehensive observation, precise dissection, and adequate hemostasis. In our study, the rate of adverse events was lower than that during STER (31.6%) 16 . Additionally, the high mobility of extraluminal SMTs poses challenges to endoscopic angles and increases the risks of tumor residue and capsule damage during EFTR or STER; conversely, dissecting from the serosal side of the abdominal cavity helps stabilize the lesion and improve the en bloc resection rate 7 .

This new technique also carries potential risks such as severe bleeding, damage to important intra-abdominal structures, difficulty in retrieving the specimen, and intraperitoneal implantation and metastasis. Therefore, the procedure should be performed by experienced endoscopic surgeons with a profound understanding of intra-abdominal structures, and only on selected patients without severe adhesions. We recommend physicians who perform this novel technique should undergo training with at least 100 cases of STER, EFTR, and peroral endoscopic myotomy, based on the known learning curve for this latter procedure 18 . Additionally, experienced surgeons and anesthesiologists should always be available as backup support.

We acknowledge several limitations to our study. First, the study included only 10 patients. Considering the low incidence of extraluminal GISTs and the fact that this is a novel technique performed by several experienced endoscopists, we believe this study represents an initial clinical experience with this innovative technique. Second, we have only performed this novel procedure in the gastric body, and it remains unclear whether other locations are also suitable for this resection approach. Third, the long-term outcomes are still unknown, and further exploration is needed to determine whether there are differences in clinical and economic value compared with surgical procedures and traditional endoscopic techniques.

In summary, our preliminary study demonstrated that EISD is a feasible and safe method for removing gastric SMTs with a predominantly extraluminal growth pattern. Further large-scale prospective studies and long-term follow-up are needed to comprehensively evaluate its safety and efficacy.
